# Supplementary material for: Evaluation of the Larvicidal Potential of the Essential Oil Pogostemon cablin (Blanco) Benth in the Control of Aedes aegypti
Source: Pharmaceuticals (Basel). 2019 Apr 8;12(2):53. doi: 10.3390/ph12020053 (PMC6630315; doi:10.3390/ph12020053)

Supplementary Materials

Evaluation of the Larvicidal Potential of the Essential Oil *Pogostemon cablin* (Blanco) Benth in the Control of *Aedes aegypti*

Figure S1. – Chromatogram of *P. cablin* essential oil.

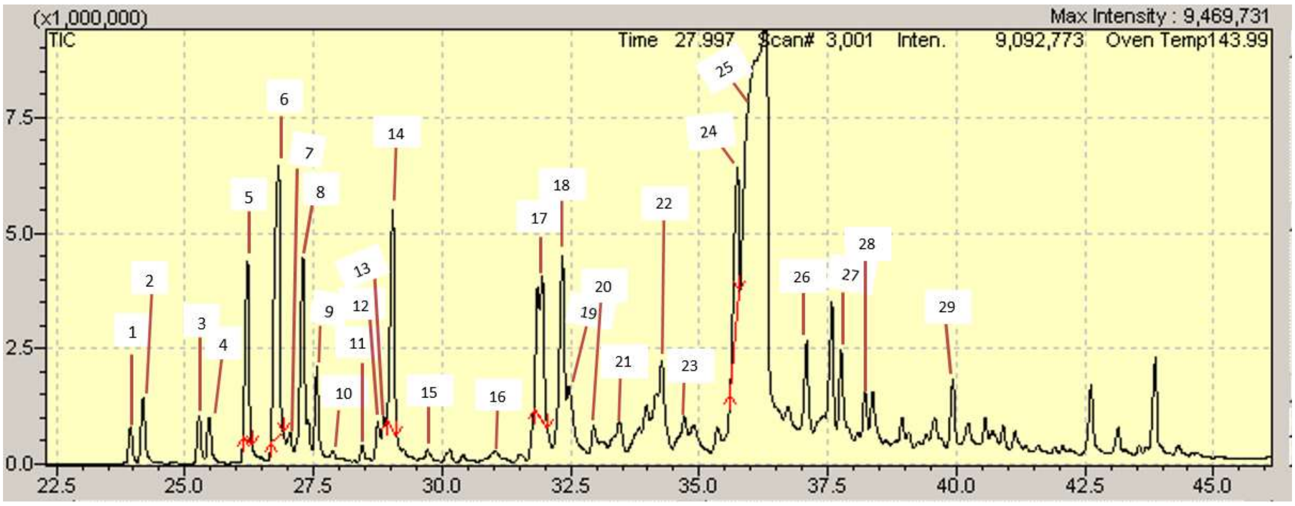

Legend: The numbers of each peak refer to the compound identified in table 1.

Figure S2 - Mass spectra of the major compounds of the essential oil of *P. cablin* by GCMS in comparison with the mass spectra of the equipment library.

Peak 6 – Substance: Seychellene (Tr 26.817 min)

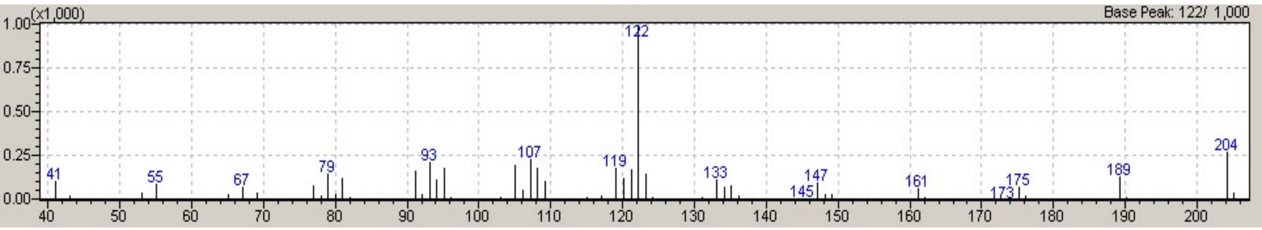

Library Spectrum

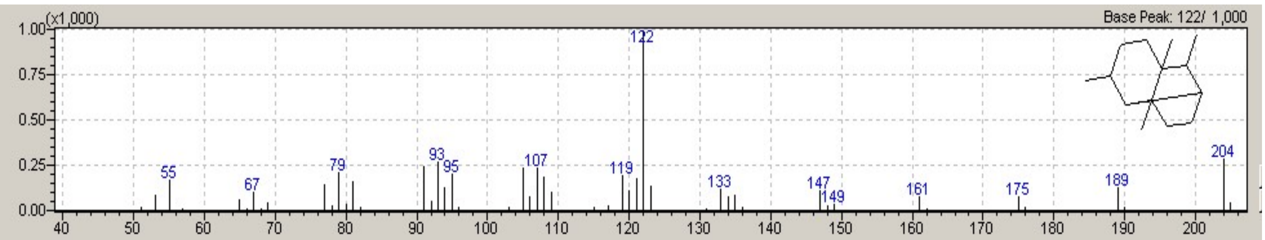

Peak 14 – Substance: α-bulnesene (Tr 29.042 min)

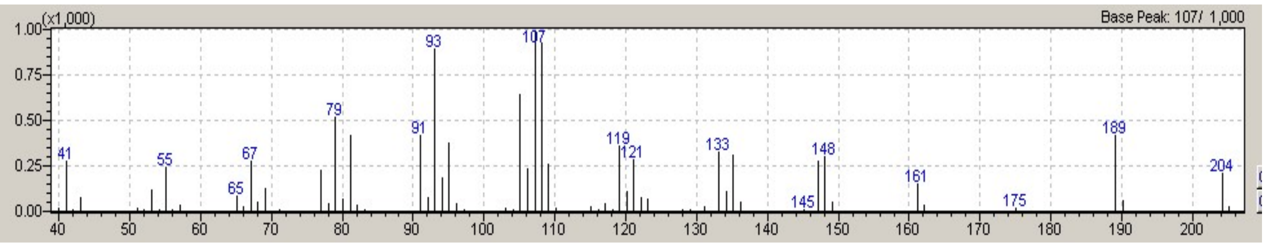

Library Spectrum

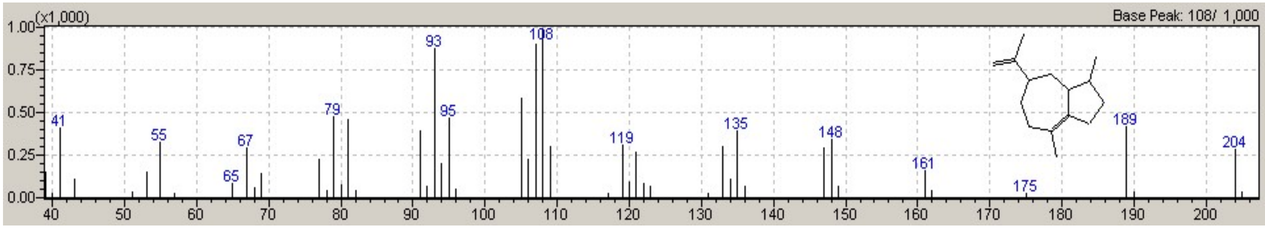

Peak 17 – Substance: Norpatchoulenol (Tr 31.942)

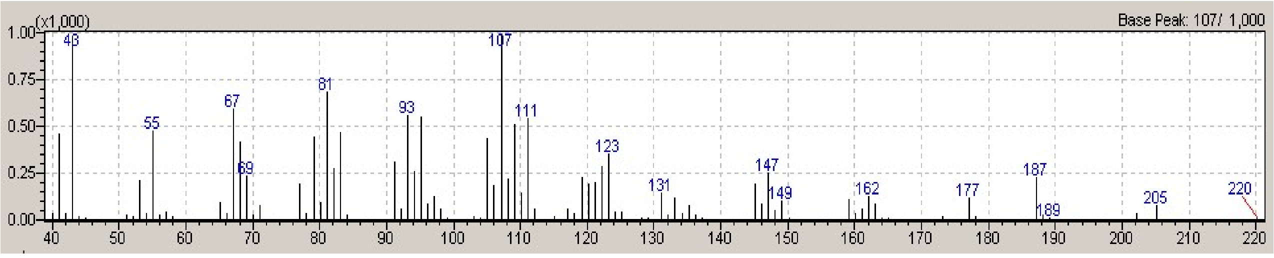

Library Spectrum

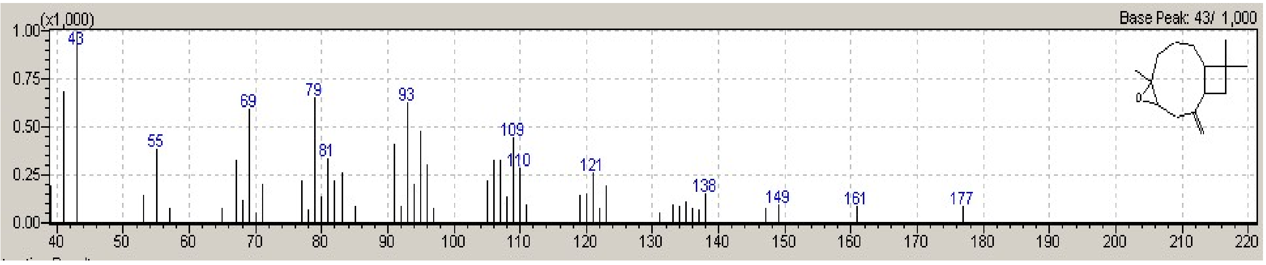

Peak 24 – Substance: Pogostol (Tr 35.742)

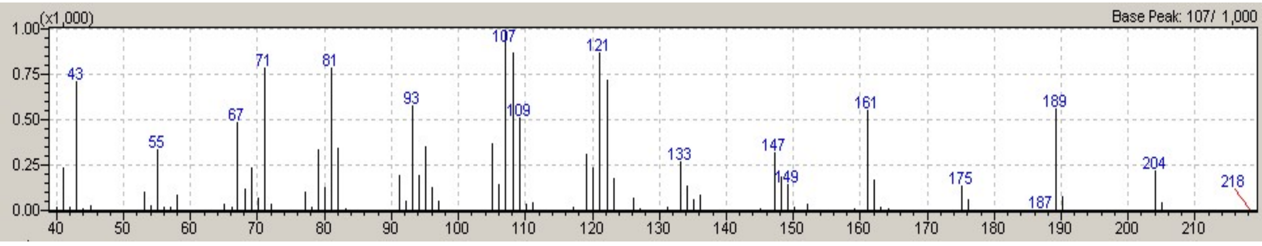

Library Spectrum

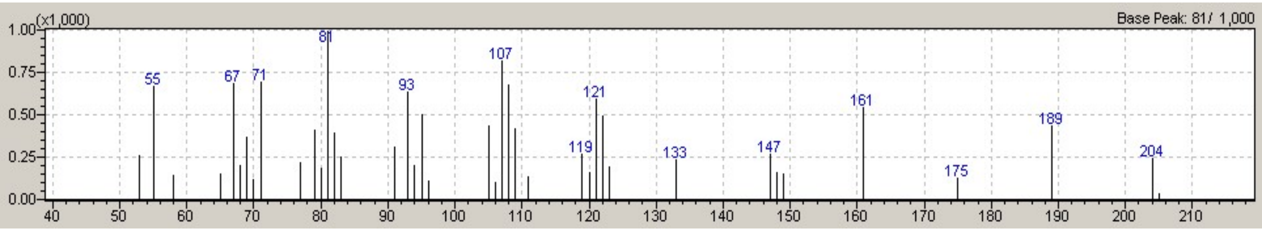

Peak 25 – Substance: Patchouli alcohol (Tr 36.308)

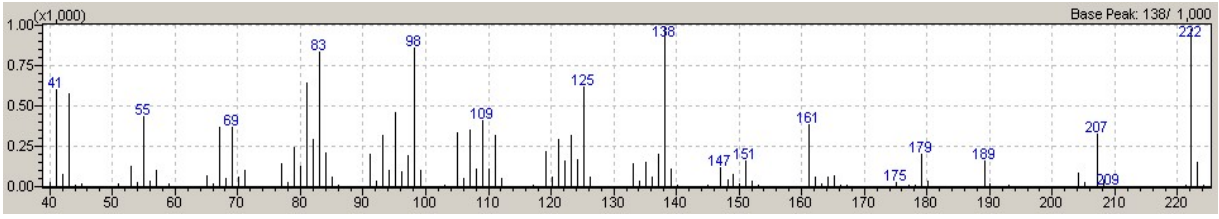

Library Spectrum

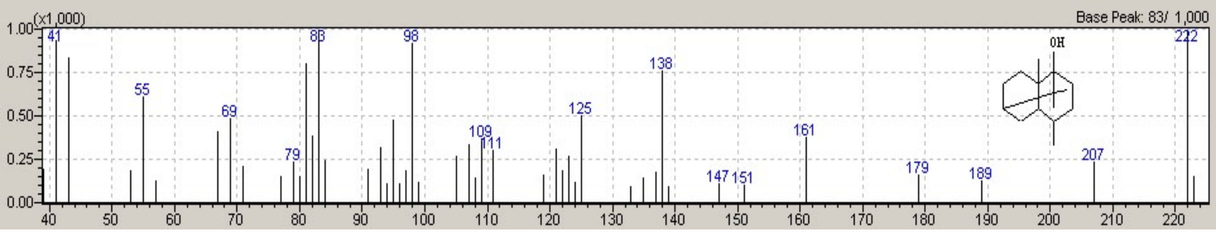

Supplement: Supplementary file 1 [file pharmaceuticals-12-00053-s001.pdf]
